# Protein Sub-Nuclear Localization Based on Effective Fusion Representations and Dimension Reduction Algorithm LDA

**DOI:** 10.3390/ijms161226237

**Published:** 2015-12-19

**Authors:** Shunfang Wang, Shuhui Liu

**Affiliations:** School of Information Science and Engineering, Yunnan University, Kunming 650504, China; zzuielsh@163.com

**Keywords:** protein sub-nuclear localization, DipPSSM, PseAAPSSM, linear discriminant analysis, KNN classifier

## Abstract

An effective representation of a protein sequence plays a crucial role in protein sub-nuclear localization. The existing representations, such as dipeptide composition (DipC), pseudo-amino acid composition (PseAAC) and position specific scoring matrix (PSSM), are insufficient to represent protein sequence due to their single perspectives. Thus, this paper proposes two fusion feature representations of DipPSSM and PseAAPSSM to integrate PSSM with DipC and PseAAC, respectively. When constructing each fusion representation, we introduce the balance factors to value the importance of its components. The optimal values of the balance factors are sought by genetic algorithm. Due to the high dimensionality of the proposed representations, linear discriminant analysis (LDA) is used to find its important low dimensional structure, which is essential for classification and location prediction. The numerical experiments on two public datasets with KNN classifier and cross-validation tests showed that in terms of the common indexes of sensitivity, specificity, accuracy and MCC, the proposed fusing representations outperform the traditional representations in protein sub-nuclear localization, and the representation treated by LDA outperforms the untreated one.

## 1. Introduction

It is well known that if proteins are wrongly located or are largely accumulated in improper parts in nuclear, genetic diseases, and even cancer, will be caused [1]. Thus, nuclear protein plays a very important role in the research on disease prevention and clinical medicine where the correct protein sub-nuclear localization is essential. Researchers in the past two decades have made great progress in the study of protein representation methods and sub-cellular localization prediction [2]. Since the nucleus is the largest cell organelle guiding the process of biological cell life, researchers have focused on seeking out the location(s) in the nucleus of the query protein so as to explore its function. The traditional approaches are to conduct a series of biology experiments at the cost of much time and money [3]. However, the task, with a large number of protein sequences having been generated, requires us to find faster localization methods. An attractive route in recent studies is to utilize machine learning for protein sub-nuclear localization [4].

The core problem of protein sub-nuclear localization using machine learning method includes two aspects: constructing good representations for collecting as much protein sequence information as possible, and developing effective models for prediction. Some good representations providing abundant discrimination information for improving prediction accuracy have been reported. Nakashima and Nishikawa propose the well-known representation, amino acid composition (AAC) [5], which describes the occurrence frequency of 20 kinds of essential amino acids in the protein sequence. However, AAC loses the abundant information of protein sequence. Then, dipeptide composition (DipC) is presented by considering the essential amino acid composition information along local order of amino acid [6]. Subsequently, taking into account both sequence order and length information, Chou *et al.* introduce pseudo-amino acid composition (PseAAC) [7,8,9,10,11]. Besides, position-specific scoring matrix (PSSM) is proposed through considering the evolution information that is helpful for protein sub-nuclear localization [12]. In addition, many representation approaches can be found in [13,14].

After obtaining a good representation, researchers need to develop models for predicting protein sub-nuclear localization. Shen and Chou [15] utilize optimized evidence-theoretic *k*-nearest classifier based on PseAAC to predict protein sub-nuclear locations. Mundra *et al.* report a multi-class support vector machine based classifier employing AAC, DipC, PseAAC and PSSM [16]. Kumar *et al.* describe a method, called SubNucPred, by combining presence or absence of unique Pfam domain and amino acid composition based SVM model [17]. Jiang *et al.* [18] report an ensemble classification method for sub-nuclear locations on dataset in [19,20] using decision trumps, Fuzzy *k*-nearest neighbors algorithm and radial basis-SVMs.

However, two drawbacks in current works exist: shortage of a representation with sufficient information and no consideration of the relationship between representation and prediction model. Using single representation, from one point of view, is insufficient for expressing protein sequence, which can lead to bad performance on protein sub-nuclear localization. Representations with more information from multiply aspects are worth studying for improving prediction accuracy. On the other hand, simplicity is also an important principle in machine learning. A compact representation can yield a preferred prediction model [21]. Therefore, this paper first proposes two effective fusion representations by combining two single representations, respectively, and then uses the dimension reduction method of linear discriminant analysis (LDA) to arrive at an optimal expression for *k*-nearest neighbors classifier (KNN). In the first process, we specifically take account into both DipC and PSSM to form a new representation, dubbed DipPSSM and consider both PseAAC and PSSM to construct another proposed representation, called PseAAPSSM. In this way, the two proposed representations contain more protein sequence information, and can be sufficient for describing protein data. However, it is difficult to reach a suitable trade-off of DipC and PSSM in DipPSSM and a suitable trade-off of PseAAC and PSSM in PseAAPSSM, so we adopt genetic algorithm to figure out a set of balance factors to solve this problem.

**Table 1:** The corresponding relationship between abbreviation and full name.

Code	The Full Name	Abbrevition
1	Dipeptide composition	DipC
2	Pseudo-amino acid composition	PseAAC
3	Position specific scoring matrix	PSSM
4	The proposed representation by fusing DipC and PSSM	DipPSSM
5	The proposed representation by fusing PseAAC and PSSM	PseAAPSSM
6	Linear discriminate analysis	LDA
7	*k*-nearest neighbors	KNN
8	True positive	TP
9	True negative	TN
10	False positive	FP
11	False negative	FN
12	Mathew’s correlation coefficient	MCC

In Section 2, we review three single representations, DIPC, PseAAC and PSSM. In Section 3, we propose two representations and use genetic algorithm to get the balance factors of the proposed representations. In Section 4, we perform LDA on the proposed representations followed by KNN classification algorithm. In Section 5, experiments with two benchmark datasets are performed. Section 6 gives the concluding remarks. For convenience of the readers, we give a list of all abbreviations of this paper in Table 1.

## 2. The Related work

In this section, three single representations, DIPC, PseAAC and PSSM, are introduced to prepare for our proposed fusion representations.

### 2.1. Dipeptide Composition (DipC)

DipC, reflecting the amino acids composition information and the ordinal relation of the essential amino acids in the sequence, denotes the occurrence frequencies of dyad consecutive residues in the primary sequence out of the 400 combination of dyad amino acids and hence forms a 400D feature vector [6]. In this work, we add 20 elements, separately representing the frequencies of 20 kinds of amino acids in the protein sequence, into DipC vector to preferably reflect the amino acids composition information. Therefore, the final protein sequence is expressed as a 420 dimensions vector that can be mapped into a point of 420D Euclidean spaces. We denote this feature representation of a protein sample as *P*_DipC_, whose former 20D shows the amino acids composition and latter 400D shows dipeptide composition. For a protein P whose sequence length is *L* (*i.e.*, P has *L* amino acids), we have

(1) $$P_{DipC}={\left[p_{1},p_{2},\cdots ,p_{20},p_{21},\cdots ,p_{420}\right]}^{T},p_{i}=\left\{ \begin{array}{ll} aa_{i}/L, & i=1,2,\cdots ,20\\ cr_{i}/\left(L-1\right), & i=21,22,\cdots ,420 \end{array}\right.$$

where *aa_i_* is the amount of type *i* amino acids and *cr_i_* is the amount of dyad consecutive residues.

### 2.2. Pseudo Amino acid Composition (PseAAC)

PseAAC, put forward by Chou *et al*., represents a protein sequence with its sequence composition and order information in a vector [7]. In PseAAC, the first 20 elements denote the frequency of 20 kinds of essential amino acids and the rest elements are the ordinal related factor obtained via computing the impact of the hydrophobic and hydrophilic of amino acids [15]. General PseAAC is written as:

(2) $$P_{PseAAC}={\left[p_{1},\cdots ,p_{20},\cdots ,p_{20+\lambda },\cdots ,p_{20+2\lambda }\right]}^{T}$$

In this paper, we transform protein sequence into PseAAC representation with tools on line provided by Pattern Recognition and Bioinformatics Group of Shanghai Jiaotong University. Note that we empirically set the value of parameter λ as 10 and obtain a 40D feature vector *P*_PseAAC_ for representing the protein sequence P.

### 2.3. Position Specific Scoring Matrix (PSSM)

There are various variations of protein sequences occurring in the biological evolution process, for instance, the insertion, substitution or deletion of one or several amino acid residues in the sequence [21]. With long-term accumulation of these variations, the similarities between the original and the new synthesis proteins are reducing gradually, but these homologous proteins may exhibit remarkably similar structures and functions [22]. As one sub-nuclear location may contain highly homologous proteins with similar biological function, we employ PSSM to collect protein sequences evolution information. Here, we obtain PSSM with the PSI-BLAST search tool provided on line by National Center for Biotechnology Information, via three iterations setting the *E*-value cutoff at 0.001 for the query sequence of the protein P against multiple sequence alignment. Then protein sequence P is represented as a matrix shown in Equation (3).

(3) $$P_{PSSM}=\left[\begin{array}{llll} p_{\left(1,1\right)}, & p_{\left(1,2\right)}, & \cdots , & p_{\left(1,20\right)}\\ p_{\left(2,1\right)}, & p_{\left(2,2\right)}, & \cdots , & p_{\left(2,20\right)}\\ \vdots & \vdots & \vdots & \vdots \\ p_{\left(L,1\right)}, & p_{\left(L,2\right)}, & \cdots , & p_{\left(L,20\right)} \end{array}\right]$$

where *P*_(*i*,*j*)_ is the score that the *i*-th amino acid is substituted by the type *j* amino acid [23], *i* = 1,2,…, *L*; *j* = 1,2,…, 20. Here, the numerical codes from 1 to 20 denote the 20 native amino acid types corresponding to their single character codes in the alphabetical order. We see that the *L* × 20 PSSM matrices are not uniform for proteins with different sequence lengths *L*, which cannot be processed by general machine learning methods. To uniform PSSM dimension, we define a new matrix *M* = *P_PSSM_*^T^*·P_PSSM_*, which is a symmetric matrix containing 20 × 20 = 400 elements [24,25]. Thus, we only need the information of its 210 elements just as Equation (4).

(4) $$\left[\begin{array}{llll} p_{\left(1,1\right)} & & & \\ p_{\left(2,1\right)} & p_{\left(2,2\right)} & & \\ \vdots & \vdots & \ddots & \\ p_{\left(L,1\right)} & p_{\left(L,2\right)} & \cdots & p_{\left(L,20\right)} \end{array}\right]\overset{\Delta }{=}\left[\begin{array}{llll} p_{1} & & & \\ p_{2} & p_{3} & & \\ \vdots & \vdots & \ddots & \\ p_{191} & p_{192} & \cdots & p_{210} \end{array}\right]$$

Then the general protein sample P can be formulated as:

(5) $$P_{PSSM}={\left[p_{1},p_{2},p_{3},\cdots ,p_{210}\right]}^{T}$$

## 3. Two Fusion Representations, DipPSSM and PseAAPSSM, and the Optimization Algorithm

In this section, two fusion representations are introduced and then genetic algorithm is used to seek out the optimal weight coefficients in the fusing process.

### 3.1. Two Fusion Representations DipPSSM and PseAAPSSM

Although both of DipC and PseAAC contain the information of the amino acid composition and the sequence order, they reflect different essential features of protein samples. On the other hand, PSSM represents a protein’s evolution information, which DipC and PseAAC do not possess. To this end, we combine PSSM with DipC and PseAAC to form two new representations, called DipPSSM and PseAAPSSM, respectively. Both DipPSSM and PseAAPSSM contain much more protein information than their component representations. Specifically, DipPSSM includes amino acids composition information, amino acids sequence order information and evolutionary information. PseAAPSSM contains amino acids composition information, amino acids sequence order information, the chemical and physical properties of amino acids and evolutionary information.

Now, we introduce the detailed combination of generating the fusion representations, DipPSSM and PseAAPSSM. Suppose that we have a dataset of *N* proteins belonging to *n* sub-nuclear locations. First, we transform the protein sequence of the *i*-th sub-nuclear location into two representations *A_i_* and *B_i_*, *i* = 1,2,…, *n*, where *A_i_* means DipC or PseAAC and *B_i_* means PSSM. *A_i_* and *B_i_* contain different context information leading to their different effects on protein sub-nuclear localization. Denote *A* and *B* as follows [7,15,24].

(6) $$A=\{A_{1},A_{2},A_{3},\cdots ,A_{n-1},A_{n}\}$$

(7) $$B=\{B_{1},B_{2},B_{3},\cdots ,B_{n-1},B_{n}\}$$

Then, we employ the weight coefficients vector *R* to balance the two representations, which is an important idea for combining representations. The mathematical forms of *R* can be written as follow:

(8) $$R=\{r_{1},r_{2},r_{3},\cdots ,r_{n-1},r_{n}\}$$

where *r_i_* ∈ (0,1) (*i* = 1,2,…, *n*) are used to represent the importance of the two representations in each sub-nuclear location, here also called the balance factors. We present the final form of the fusion representation in Equation (9).

(9) $$V_{i}=[r_{i}A_{i},(1-r_{i})B_{i}]\;(i=1,2,\cdots ,n)$$

In many current literatures, different components of a fusion representation are considered equally important, which is actually a special case of Equation (9) when *r_i_* = 0.5 (*i* = *1*,*2*,…, *n*). Since the fused representation Equation (9) uses the characteristics of the two single representations reasonably, it contains more protein sequences information and reflects the influence degree of the two single representations. Note that the balance factors for different sub-nuclear locations are not all the same. Besides, since different sub-nuclear locations are an organic whole in the cellular nucleus, the sub-nuclear proteins are interacting with each other, it is proper to think that *n* balance factors, *r_i_* (*i* = 1,2,…, *n*), are correlated with each other. Therefore, it is a complex work to select an optimum value of *R*. In the next subsection, we will discuss how to give a proper value of *R.*

### 3.2. Genetic Algorithm—The Optimization Algorithm

Genetic algorithm is an algorithm that imitates the evolution process of biological organism in the nature as an adaptive method that can be used to solve searching and optimizing problems [26], especially combination optimization problems with high computational complexity, which traditional methods cannot cope with [27]. In this paper, we employ genetic algorithm to seek out the balance factors *r_i_* (*i* = 1,2,…, *n*) of the proposed representations. The seeking procedure is as follows.

The first and generally the most difficult step of the genetic algorithm is to create an initial population, which is a pre-determined amount of individuals encoded to map the problem solution into a genetic string, or chromosome [28]. In genetic algorithm, all the individuals, in term of the coding method and principle, possess the same structure maintaining the genetic information on individuals of population. The second step is to conduct selection, crossover, mutation and replacement depending on the fitness error, under the constraints of the individual population. The final step is to stop iteration when stopping criteria is met.

In this paper, we put forward an initial-population selection strategy to greedily produce initial population. Its detailed process is as follows.

(1) Generate a random permutation of the integers traversing from 1 to *n* (*n* is the number of sub-nuclear locations), which is the tuning order of the balance vector *R*.
(2) Set 0.5 as the initial value for all elements in *R*.
(3) For each *r_i_*, we search from 0 to 1 with 0.01 steps to get the value obtaining the highest prediction accuracy.
(4) Repeat step (3) for all the elements of *R* according to the order in step (1).
(5) Repeat step (1–4) 50 times to get 50 sets of balance vectors *R*. We save these balance vectors as the initial population.

Note that in Step (5), due to the unstable of genetic algorithm, we here run this experiment multiple times to select the optimal solution as the final balance factors. Specifically, we repeat 50 times to generate an initial population. In theory, the greater the number of repetitions, the better the result becomes. Practically, the results trend to be stable when the repetition exceeds 50 times. Therefore, we set a relative reasonable number of 50 due to the cost of computation. After the steps for creating the initial population, we calculate the balance factors via minimizing the fitness error for predicting the sub-nuclear localization. We implement the computation by using MATLAB to work out the balance factors *r_i_* (*i* = 1,2,…, *n*) delivering by the minimum fitness error.

## 4. Dimension Reduction Method and Classifier Algorithm

In this section, we first introduce the dimension reduction algorithm and then describe the KNN classifier and cross-validation methods.

### 4.1. Linear Discriminant Analysis (LDA)

It is well known that high dimension of data not only increases the complexity of classifier, but also increases the risk of over fitting of the classifier [12]. The increase in information and dimensionality of our proposed fusing representations will lead to an increase in noise [29]. Specifically, each representation has its intrinsic dimensionality for classification which is often much lower than the dimensionality of the observation vector. Hence, the dimensionality reduction algorithm, linear discriminant analysis (LDA) [22,30], is employed in this work, which is a well-known supervised classifier in pattern recognition such as speech recognition, face recognition, protein classification and so on. A concise description about LDA is given below.

Assume that Dataset *X* contains *N* proteins and *X* is a union of *C* subsets, *i.e.*, $X=X_{1}\cup X_{2}\cup \cdots X_{C}=\left\{x_{1},x_{2},\cdots x_{N}\right\}$, where *X_i_* contains *N*(*i*) proteins $x_{1}^{i},x_{2}^{i},\cdots ,x_{N\left(i\right)}^{i}$, *i* = 1,2,…, *C*. Thus, $N=\sum_{i=1}^{C}N\left(i\right)$. Suppose $X_{i}\cap X_{j}=\varphi ,i,j=1,2,\cdots ,C,i\neq j$. To obtain the optimal solution of LDA, we maximize the formulation *J*(*W*) in Equation (10) and then find out the projection matrix W*. We can realize the ideal linear projection with the projection matrix W*.

(10) $$J(W)=\frac{|W^{T}S_{B}W|}{|W^{T}S_{W}W|},W^{*}=\underset{W}{argmax}J(W)$$

where *S_W_* and *S_B_* denote within-class scatter matrix and between-class scatter matrix, respectively, which are formulized as follows.

(11) $$S_{W}=\sum_{i=1}^{C}\sum_{j=1}^{N_{i}}\left(x_{j}^{i}-\mu_{i}\right){\left(x_{j}^{i}-\mu_{i}\right)}^{T}$$

(12) $$S_{B}=\sum_{i=1}^{C}N_{i}(\mu_{i}-\mu ){\left(\mu_{i}-\mu \right)}^{T}$$

where $\mu_{i}=\frac{1}{N_{i}}\sum_{j=1}^{N_{i}}x_{j}^{i}$ is the class mean vector and $\mu =\frac{1}{N}\sum_{i=1}^{C}N_{i}\mu_{i}$ is the total mean vector.

For the focus of this paper, we do not give too many descriptions for the derivation and calculation process of matrix W*. According to [31,32], for multi-class pattern classification, such as *C* classification problem, the orthonormal columns of W^*^ must satisfy Equation (13), which is a generalized eigenvalue problem.

(13) $$S_{B}w_{i}=\lambda_{i}S_{W}w_{i},\;i=1,2,\cdots ,C-1$$

Hence, the eigenvectors of ${S_{W}}^{-1}S_{B}$ consistent with the largest *C* − 1 eigenvalues are the columns of the optimal projection matrix W* on the condition that *S_W_* is nonsingular.

Finally, we obtain the projection $Y=(y_{1},y_{2},\cdots ,y_{C-1})$ through Equation (14):

(14) $$Y={\left(W^{*}\right)}^{T}X$$

### 4.2. k-Nearest Neighbors (KNN) Algorithm and Cross-Validation Methods

#### 4.2.1. *k*-Nearest Neighbors Algorithm

For protein sub-nuclear localization and classification problem, one classic and simple method is *k*-nearest neighbors (KNN). The KNN classifier predicts each unlabeled sample by the majority label of its nearest neighbors in the training set [33]. Despite its simplicity, the KNN often yields competitive results, and in this paper, when combined with the reduction dimension algorithm, it has significantly advanced the classification accuracy [23]. Before applying KNN classifier for protein sub-nuclear localization, we transform each protein sequence to a vector with fixed dimension. Then we classify each sequence according to class memberships of its *k*-nearest neighbors [34,35]. Cosine distance, $cos(u,v)=\frac{u\cdot v}{\left\|u\right\|\times \left\|v\right\|}$, is chosen to measure the close degree of two proteins *u* and *v*, where ‖⋅‖ is the module function. The value of *cos*(*u*, *v*) ranges in [–1,1], the closer to 1 its absolute value is, the closer to each other are *u* and *v*.

#### 4.2.2. Cross-Validation Methods

Traditionally, in the context of statistical prediction and classification, cross-validation is utilized to estimate the performance of the final classifier or predictor. Independent dataset test, jackknife test, and *K*-fold cross-validation are three popular cross validation methods [35]. The *K*-fold cross-validation is a method to approximately estimate prediction error without bias under much more complicated situations [36]. Thus *K*-fold cross-validation is employed in this paper to examine the anticipated performance of the KNN classifier, where *K* is the positive integer satisfying *K* ≤ *N* and *N* denotes the size of the benchmark dataset. The case *K* = *N* is indeed identify to leave-one-out or jackknife test. Jackknife test can deliver high variance on account of the *N* training sets similar to one another [37]. Moreover, the computational cost is also expensive, requiring *N* iterations of the learning approach. Usually, 10-fold cross validation is a preferred route for pursuing a good trade off, where the benchmark dataset is randomly partitioned into ten equal-size subsets where those subsets hold the original proportion in different classes. For each experiment, we carry out the test ten times. In each run, one subset is utilized for testing and the remaining are used for training, and thus each subset is in turn used as testing set once. To obtain a reliable result, we run 50 times experiments and calculate the average result of the test accuracies. In addition, since the jackknife test is objective and little arbitrary because it can always yield a unique result for a given dataset, and therefore has been adopted to estimate the performance of predictors [38], it is also considered in Section 5.2.4 to compare the overall success rate of predictors.

## 5. Numerical Results

In this section, we introduce the two sub-nuclear location datasets and then give the numerical results and analysis.

### 5.1. Description of Datasets and Experimental Procedure

In order to validate the efficiency of the proposed method, two public datasets are adopted in this paper. One is Nuc-Ploc [7], constructed in 2007 by Shen and Chou, which contains 714 proteins located at nine sub-nuclear locations, listed in Table 2. The other is SubNucPred [17], constructed by Ravindra Kumar *et al.* in 2014, which contains ten sub-nuclear location proteins and is detailed listed in Table 3.

**Table 2:** Protein benchmark Dataset 1 of nine sub-nuclear locations.

Code	Sub-Nuclear Location	Number
1	Chromatin	99
2	Heterochromatin	22
3	Nuclear envelope	61
4	Nuclear matrix	29
5	Nuclear pore complex	79
6	Nuclear speckle	67
7	Nucleolus	307
8	Nucleoplasm	37
9	Nuclear PML body	13
Overall		714

**Table 3:** Protein benchmark Dataset 2 of ten sub-nuclear locations.

Code	Sub-Nuclear Location	Number
1	Centromere	86
2	Chromosome	113
3	Nuclear speckle	50
4	Nucleolus	294
5	Nuclear envelope	17
6	Nuclear matrix	18
7	Nucleoplasm	30
8	Nuclear pore complex	12
9	Nuclear PML body	12
10	Telomere	37
Overall		669

The procedure of numerical experiment is as follows.

(1) Represent the protein sequences using DipC, PseAAC, and PSSM.
(2) Fuse DipC and PSSM to get DipPSSM and fuse PseAAC and PSSM to get PseAAPSSM.
(3) Employ LDA to reduce the dimensionality of DipPSSM and PseAAPSSM.
(4) Train KNN classifier for prediction.

**Figure 1:**
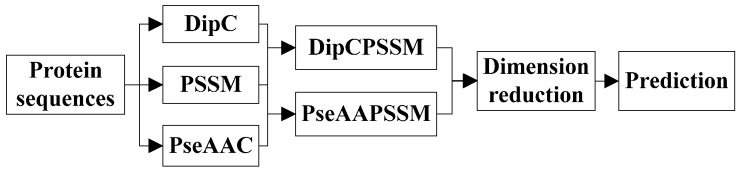
A flowchart of the prediction process.

To provide an intuitive view, these processes are shown in Figure 1.

### 5.2. Numerical Results and Analysis

#### 5.2.1. Feature Fusion Representations

A comparison of fusing and single representations: In this subsection, we compare our proposed representations PseAAPSSM and DipPSSM with their single atoms on the prediction success rates of protein sub-nuclear locations. Table 4 and Table 5 show the experimental results for every sub-nuclear location on Datasets 1 and 2, respectively. Note that we take the average value of fifty random success rates according to 10-fold cross validation as the prediction success rate (SR), where the neighborhood size *k* of KNN is chosen corresponding to the highest overall success rate with *k* traversing from 1 to 10. The calculation of success rate and overall success rate are in Equations (15) and (16), respectively.

(15) success rate(i)=T(i)/N(i)   (i=1,2,⋯,n)

(16) $$overall\;success\;rate\;=\sum_{i=1}^{n}T(i)/\sum_{i=1}^{n}N(i)\;(i=1,2,\cdots ,n)$$

where *T*(*i*) is the number of correctly predicted proteins belonging to location *i*, *N*(*i*) is the total number of proteins at location *i*. Note that the success rate here can also be understood as the sensitivity defined in many literatures which will be discussed in Section 5.2.3. For the two proposed fusion representations DipPSSM and PseAAPSSM, the optimal balance factor vector *R* is also listed in the tables.

According to Table 4 and Table 5, it is clear that our proposed fusion representations outperform the single representations consistently.

**Table 4:** Prediction success rate (SR) and the optimal *R* of Dataset 1 for protein sub-nuclear localization by 10-fold cross validation with various representations.

Sub-Nuclear Location	PseAAC	DipC	PSSM	PseAAPSSM		DipPSSM	
	SR (*k* = 9)	SR (*k* = 8)	SR (*k* = 3)	SR (*k* = 3)	*R*	SR (*k* = 3)	*R*
1. Chromatin	0.4867	0.5437	0.5690	0.7622	0.7500	0.7683	0.7470
2. Heterochromatin	0.2130	0.2113	0.4020	0.5650	0.8219	0.5613	0.8196
3. Nuclear envelope	0.2678	0.2169	0.3872	0.4657	0.2500	0.4530	0.2458
4. Nuclear matrix	0.1333	0.1567	0.3850	0.7777	0.9978	0.8007	0.9976
5. Nuclear pore complex	0.5480	0.5760	0.6108	0.7251	0.1500	0.7231	0.1489
6. Nuclear speckle	0.2926	0.3355	0.3303	0.5216	0.0600	0.5235	0.0583
7. Nucleolus	0.7952	0.7713	0.7756	1.0000	0.9989	1.0000	0.9997
8. Nucleoplasm	0.0577	0.0700	0.2937	0.7032	0.9978	0.7553	0.9973
9. Nuclear PML body	0.0830	0.0920	0.3820	0.4130	0.0400	0.3830	0.0401
Overall	0.5365	0.5389	0.5929	0.7971	–	0.8002	–

**Table 5:** Prediction success rate (SR) and the optimal *R* of Dataset 2 for protein sub-nuclear localization by 10-fold cross validation with different representations.

Sub-Nuclear Location	PseAAC	DipC	PSSM	PseAAPSSM		DipPSSM	
	SR (*k* = 9)	SR (*k* = 9)	SR (*k* = 6)	SR (*k* = 4)	*R*	SR (*k* = 4)	*R*
1. Centromere	0.2495	0.0916	0.6088	0.7908	0.9911	0.7889	0.9901
2. Chromosome	0.3397	0.3861	0.4819	0.9299	0.9976	0.9279	0.9980
3. Nuclear speckle	0.3188	0.3164	0.3504	0.3460	0.6983	0.3416	0.7000
4. Nucleolus	0.8679	0.8692	0.8301	0.9360	0.2504	0.9337	0.2498
5. Nuclear envelope	0.2670	0.0980	0.0070	0.0640	0.1978	0.0060	0.2000
6. Nuclear matrix	0.1880	0.1660	0.2630	0.3110	0.2391	0.3170	0.2400
7. Nucleoplasm	0.0313	0.0307	0.1667	1.0000	0.9992	1.0000	0.9998
8. Nuclear pore complex	0.4110	0.4750	0.3210	0.5080	0.2187	0.5190	0.2206
9. Nuclear PML body	0.0010	0.0020	0.0260	0.0850	0.2079	0.0660	0.2100
10. Telomere	0.0998	0.0873	0.3923	0.4738	0.1213	0.4725	0.1200
Overall	0.5168	0.5025	0.5931	0.7874	–	0.7855	–

**Figure 2:**
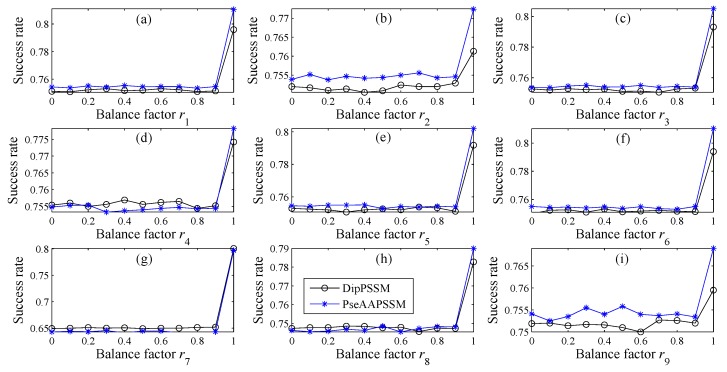
Success rate comparison for different *r_i_* with our representations on Dataset 1, where each subplot, from (**a**) to (**i**), respectively represents each sub-nuclear location.

Balance factor vector *R*: Figure 2 describes the success rate curves on Dataset 1 of DipPSSM and PseAAPSSM, where each subplot corresponds to a sub-nuclear location. For each subplot, the horizontal axis represents certain balance vector *r_i_* and the ordinate axis is the prediction success rate. Note that in each subplot, when *r_i_* varies from 0 to 1 with step 0.1, the remaining *n*−1 balance factors are fixed in the values in Table 4.

**Figure 3:**
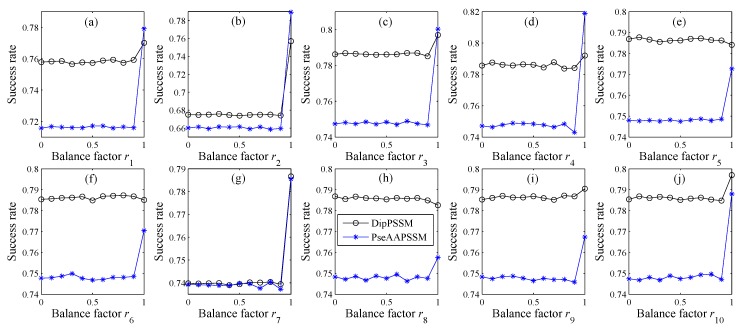
Success rate comparison for different *r_i_* with our representations on Dataset 2, where each subplot, from (**a**) to (**j**), respectively represents each sub-nuclear location.

The numerical experiment shown in Figure 3 is the same as that on Figure 2, except for the different Dataset 2. From Figure 2 and Figure 3, it is clear that the parameters *r_i_* (*i* = 1, 2,…, *n*) have significant influence on protein sub-nuclear localization. Especially, Figure 2 also shows that when *r_i_* is around 0.9 for each subplot (*i* = 1, 2,…, *n*), the success rates have a leaping point, probably suggesting that for Dataset 1, dipeptide composition or pseudo amino acid composition are more important than position specific scoring matrix in the fusion representations.

#### 5.2.2. Dimensionality Reduction

**Figure 4:**
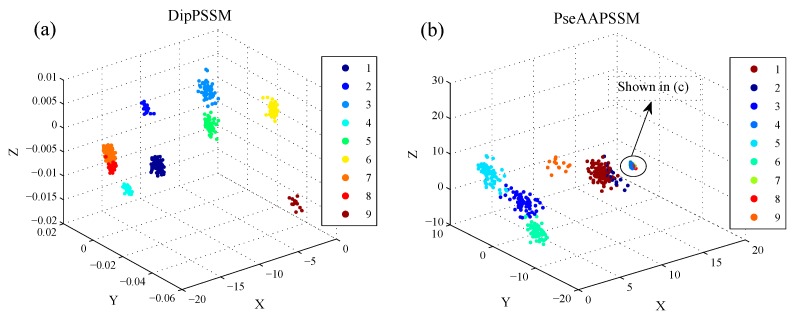

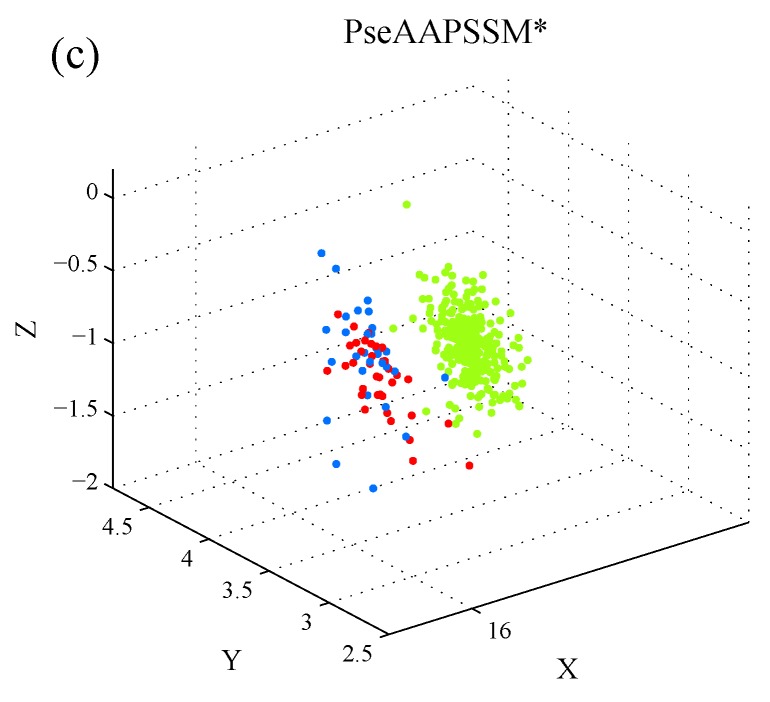
3D scatter on Dataset 1 with X-, Y- and Z-axes representing the first three components of LDA, respectively: (**a**) DipPSSM; (**b**) PseAAPSSM and (**c**) the patch of high resolution for the indicated region in (**b**).

**Figure 5:**
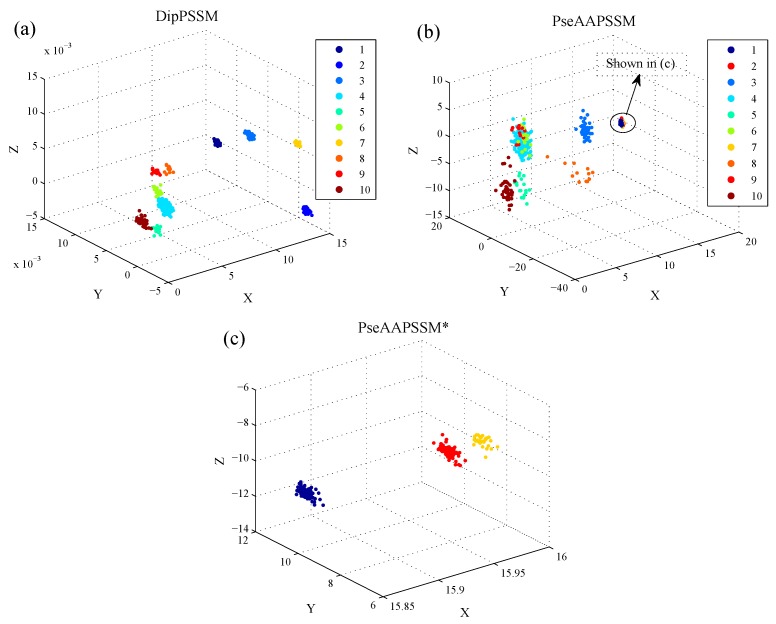
3D scatter on Dataset 2 with X-, Y- and Z-axes representing the first three components of LDA, respectively: (**a**) DipPSSM; (**b**) PseAAPSSM and (**c**) the patch of high resolution for the indicated region in (**b**).

3D visualization: In this subsection, we employ LDA to present visualization results. Here, we give the 3D scatter plot of DipPSSM and PseAAPSSM for both datasets, so as to observe the data distribution in the three-dimensional space after data reduction by LDA. Figure 4 and Figure 5 show the results of Dataset 1 and 2, respectively, where the three axes represent the first three components of LDA corresponding to the largest three eigenvalues, respectively.

In Figure 4, we use nine colors, which are coded from 1 to 9 according to Table 2, to represent the nine sub-nuclear locations protein of Dataset 1. In Figure 5, we use ten colors, which are coded from 1 to 10 according to Table 3, to represent the ten sub-nuclear locations protein of Dataset 2. In Figure 4b and Figure 5b, there are some data points that are hardly distinguished at those scales. Therefore, we provide a patch of high resolution in Figure 4c and Figure 5c for those data points. These results suggest that LDA can improve the classification performance by separating the data points from different classes.

**Figure 6:**
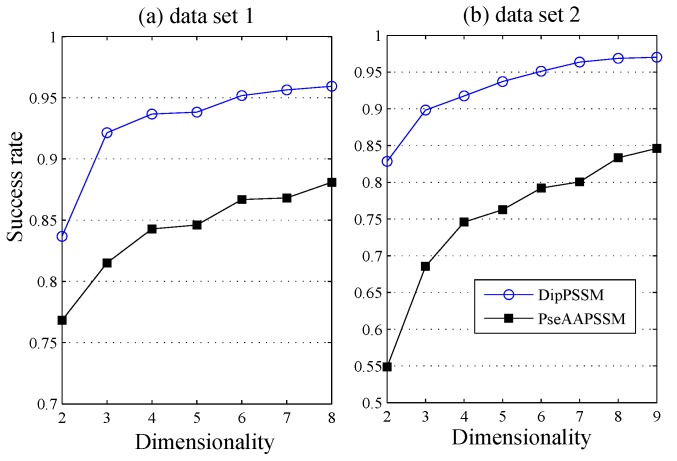
The overall success rates at different dimensions, reduced by LDA, from DipPSSM and PseAAPSSM, respectively: (**a**) Dataset 1 and (**b**) Dataset 2.

Parameter effects: With the 10-fold cross-validation, Figure 6 demonstrates the overall success rates against dimensions reduced by LDA from DipPSSM and PseAAPSSM, respectively, where the neighborhood size *k* is set to 4, a choice corresponding to a good performance in 1 to 10. From Figure 6, we can see that most information lying in the original high dimensional protein data can be summarized by some low dimensional structure, suggesting the efficiency of LDA for protein sub-nuclear localization.

**Figure 7:**
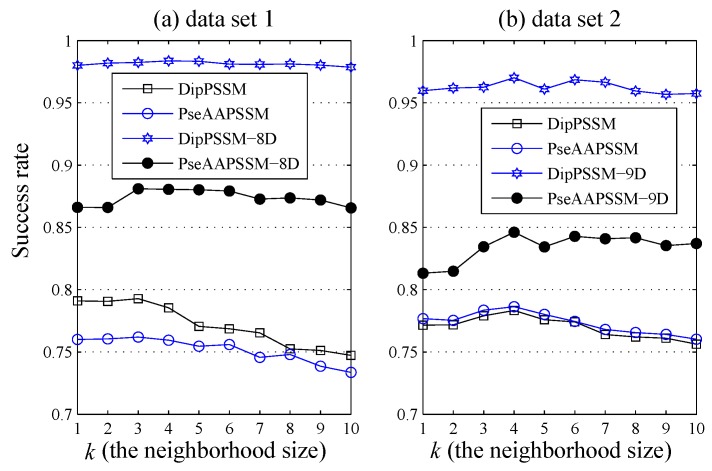
Comparison of success rates among different *k* values by DipPSSM, PseAAPSSM, DipPSSM with LDA and PseAAPSSM with LDA, respectively: (**a**) Dataset 1 and (**b**) Dataset 2.

Figure 7 further gives the comparison of the success rates among the reduction data and the original data when the neighborhood size *k* changes from 1 to 10. It is easily seen from Figure 7 that for each fixed *k*, both DipPSSM with LDA and PseAAPSSM with LDA improved success rate of sub-nuclear locating prediction significantly compared with DipPSSM and PseAAPSSM. Interestingly, in Figure 4, Figure 5, Figure 6 and Figure 7, we can see that for both datasets, the reduction effects of DipPSSM seem a little better than PseAAPSSM.

#### 5.2.3. Analysis of numerical Results

From another perspective, it is indicated in the current literature that the following indexes (Equations (17)–(20)) are often used to evaluate the performance of a predictor. We calculate these indexes of 10-fold cross validation to compare different representations together with dimension reduction method.

(17) SE(i)=TP(i)/(TP(i)+FN(i))   (i=1,2,⋯,n)

(18) SP(i)=TN(i)/(TN(i)+FP(i))   (i=1,2,⋯,n)

(19) $$ACC(i)=\frac{\left(TP(i)+TN(i)\right)}{\left(TP(i)+FP(i)+TN(i)+FN(i)\right)}\;(i=1,2,\cdots ,n)$$

(20) $$MCC(i)=\frac{\left(TP(i)\times TN(i))-(FP(i)\times FN(i)\right)}{\sqrt{\left(TP(i)+FP(i))\times (TP(i)+FN(i))\times (TN(i)+FP(i))\times (TN(i)+FN(i)\right)}}\;(i=1,2,\cdots ,n)$$

In these equations, TP (true positive) and TN (true negative) were the number of proteins that were correctly located while FP (false positive) and FN (false negative) were the number of proteins that were wrongly located. SE (Sensitivity) denotes the rate of positive samples correctly located, whose value is equal to the success rate in Equation (14). SP (Specificity) denotes the rate of negative samples correctly located. ACC (Accuracy) means the rate of correctly located samples. MCC is the Mathew’s Correlation Coefficient, which returns a value lying in [–1,1]. The value of a MCC coefficient reflects the prediction consequences. The value of 1 denotes a perfect prediction, 0 represents random prediction and −1 represents a bad prediction. We cannot perfectly describe the confusion matrix of true and false, positives and negatives through a single number, generally regarding the MCC as one of the best [39].

Table 6 gives the values of four indexes in Equations (17)–(20) for nine sub-nuclear locations in Dataset 1 using three single representations of PseAAC, Dipe and PSSM, two fusion representations of DipPSSM and PseAAPSSM and their combination with the dimension reduction method LDA, where both PseAAPSSM and DipPSSM are reduced to eight dimensions. Table 7 uses the similar experimental design to Table 6 except for the use of Dataset 2, where PseAAPSSM and DipPSSM are reduced to nine dimensions. From these results, we come to the following conclusions. The predictions with sensitivity (SE), specificity (SP), accuracy (ACC) and MCC by fusion representations are better than the single representations in most locations. Furthermore, the fusion representations with the LDA treatment outperform those without. Note that due to the randomness of the 10-fold cross validation algorithm, the numerical values of the four indexes SE, SP, ACC and MCC have small variation each time. That is also the reason why we get different values of sensitivity and success rate in each sub-nuclear location in Table 4 and Table 6, as well as Table 5 and Table 7, although theoretically Equations (15) and (17) should produce the same value.

**Table 6:** Performance of various representations on Dataset 1.

Sub-Nuclear Location	Index	PseAAC	DipC	PSSM	PseAAPSSM	DipPSSM	PseAAPSSM with LDA	DipPSSM with LDA
1. Chromatin	SE	0.4545	0.5354	0.5556	0.7475	0.8081	0.9293	0.8889
	SP	0.8472	0.8488	0.9154	0.9252	0.9154	0.9919	0.9789
	ACC	0.7927	0.8053	0.8655	0.9762	0.9006	0.9832	0.9664
	MCC	0.2633	0.3291	0.4560	0.6217	0.6441	0.9291	0.8605
2. Heterochromatin	SE	0.2727	0.1364	0.4091	0.5909	0.5909	0.5455	1
	SP	0.9884	0.9928	0.9812	0.9812	0.9855	0.9957	0.9971
	ACC	0.9664	0.9664	0.9636	0.9608	0.9734	0.9818	0.9972
	MCC	0.3255	0.2120	0.3903	0.5278	0.5642	0.6520	0.9560
3. Nuclear envelope	SE	0.2623	0.2131	0.3443	0.4754	0.4590	0.9508	0.9344
	SP	0.9893	0.9893	0.9709	0.9470	0.9770	1	1
	ACC	0.9272	0.9230	0.9174	0.9538	0.9328	0.9958	0.9944
	MCC	0.3983	0.3429	0.3831	0.5116	0.5123	0.9729	0.9637
4. Nuclear matrix	SE	0.1379	0.2069	0.4138	0.6552	0.6027	0.3793	0.5517
	SP	0.9927	0.9942	0.9737	0.9869	0.9912	0.9869	0.9839
	ACC	0.9580	0.9622	0.9510	0.9230	0.9762	0.9622	0.9964
	MCC	0.2311	0.3377	0.3813	0.6529	0.6702	0.4381	0.5543
5. Nuclear pore complex	SE	0.5316	0.5949	0.6456	0.7215	0.7342	1	1
	SP	0.9370	0.9323	0.9496	0.9622	0.9606	1	1
	ACC	0.8922	0.8950	0.9160	0.9356	0.9356	1	1
	MCC	0.4611	0.4983	0.5825	0.6763	0.6800	1	1
6. Nuclear speckle	SE	0.2985	0.3582	0.3284	0.4925	0.5075	1	1
	SP	0.9737	0.9675	0.9536	0.9675	0.9691	1	1
	ACC	0.9104	0.9104	0.8950	0.9734	0.9258	1	1
	MCC	0.3581	0.3909	0.3164	0.5074	0.5256	1	1
7. Nucleolus	SE	0.7915	0.7752	0.7590	0.9772	0.9967	0.9349	1
	SP	0.6216	0.6536	0.7125	0.9361	0.9730	0.8649	0.9926
	ACC	0.6947	0.7059	0.7325	0.9314	0.9832	0.8950	0.9958
	MCC	0.4117	0.4254	0.4669	0.9077	0.9662	0.7925	0.9915
8. Nucleoplasm	SE	0.0541	0.0811	0.2703	0.3784	0.6757	0.2703	0.9730
	SP	0.9852	0.9867	0.9838	0.9926	0.9941	0.9808	1
	ACC	0.9370	0.9398	0.9468	0.9692	0.9776	0.9440	0.9986
	MCC	0.0677	0.1169	0.3333	0.5110	0.7521	0.3152	0.9857
9. Nuclear PML body	SE	0.0769	0.1538	0.3077	0.3846	0.3077	1	1
	SP	1	0.9971	0.9929	0.9872	0.9929	1	1
	ACC	0.9832	0.9818	0.9804	0.9006	0.9804	1	1
	MCC	0.2750	0.2705	0.3602	0.3585	0.3602	1	1

**Table 7:** Performance of various representations on Dataset 2.

Sub-Nuclear Location	Index	PseAAC	Dipe	PSSM	PseAAPSSM	DipPSSM	PseAAPSSM with LDA	DipPSSM with LDA
1. Centromere	SE	0.2209	0.1163	0.5930	0.8023	0.8256	0.6163	1
	SP	0.9705	0.9828	0.9314	0.9743	0.9760	0.9674	0.9949
	ACC	0.8744	0.8714	0.8879	0.9522	0.9567	0.9223	0.9955
	MCC	0.2845	0.1948	0.5120	0.7844	0.8056	0.6304	0.9805
2. Chromosome	SE	0.3363	0.3805	0.5044	0.9027	0.8850	0.8761	1
	SP	0.8867	0.8525	0.9047	0.9910	0.9874	0.9011	1
	ACC	0.7937	0.7728	0.8371	0.9761	0.9701	0.8969	1
	MCC	0.2333	0.2240	0.4135	0.9135	0.8917	0.6917	1
3. Nuclear speckle	SE	0.2600	0.3400	0.3600	0.3200	0.3000	0.7800	1
	SP	0.9774	0.9709	0.9645	0.9742	0.9758	0.9742	1
	ACC	0.9238	0.9238	0.9193	0.9253	0.9253	0.9596	1
	MCC	0.3172	0.3672	0.3599	0.3625	0.3504	0.7220	1
4. Nucleolus	SE	0.8810	0.8707	0.8231	0.9422	0.9422	0.9320	0.9830
	SP	0.4427	0.4907	0.6480	0.8000	0.8053	0.9867	0.9840
	ACC	0.6353	0.6577	0.7250	0.8625	0.8655	0.9626	0.9836
	MCC	0.3504	0.3809	0.4710	0.7377	0.7428	0.9247	0.9666
5. Nuclear envelope	SE	0.2941	0.1176	0.0017	0.0588	0.1176	1	0.8235
	SP	0.9939	0.9954	0.9939	0.9954	0.9939	1	0.9939
	ACC	0.9761	0.9731	0.9686	0.9716	0.9716	1	0.9895
	MCC	0.3934	0.2066	−0.0125	0.1107	0.1861	1	0.7950
6. Nuclear matrix	SE	0.1111	0.1667	0.2222	0.3889	0.3333	0.8889	0.8333
	SP	0.9985	0.9985	0.9954	0.9892	0.9908	0.9985	0.9969
	ACC	0.9746	0.9761	0.9746	0.9731	0.9731	0.9955	0.9925
	MCC	0.2654	0.3466	0.3460	0.4275	0.3951	0.9124	0.8537
7. Nucleoplasm	SE	0.0016	0.0011	0.1667	0.9667	0.9667	0.2333	1
	SP	0.9969	0.9984	0.9969	0.9937	0.9906	0.9890	1
	ACC	0.9522	0.9537	0.9596	0.9925	0.9895	0.9552	1
	MCC	−0.0119	−0.0084	0.3326	0.9179	0.8898	0.3215	1
8. Nuclear pore complex	SE	0.4167	0.5000	0.2500	0.5000	0.5000	1	1
	SP	1	0.9939	0.9924	0.9939	0.9939	1	1
	ACC	0.9895	0.9851	0.9791	0.9851	0.9851	1	1
	MCC	0.6421	0.5402	0.2960	0.5402	0.5402	1	1
9. Nuclear PML body	SE	0.0020	0.0012	0.0011	0.0833	0.0833	1	1
	SP	0.9924	0.9985	0.9985	0.9954	0.9954	1	1
	ACC	0.9746	0.9806	0.9806	0.9791	0.9791	1	1
	MCC	−0.0117	−0.0052	−0.0052	0.1356	0.1356	1	1
10. Telomere	SE	0.1351	0.1351	0.4324	0.4865	0.4595	1	0.7568
	SP	0.9873	0.9747	0.9826	0.9826	0.9794	1	0.9921
	ACC	0.9402	0.9283	0.9522	0.9552	0.9507	1	0.9791
	MCC	0.2028	0.1440	0.4820	0.5265	0.4847	1	0.7904

#### 5.2.4. Compare with Existing Prediction Results

**Figure 8:**
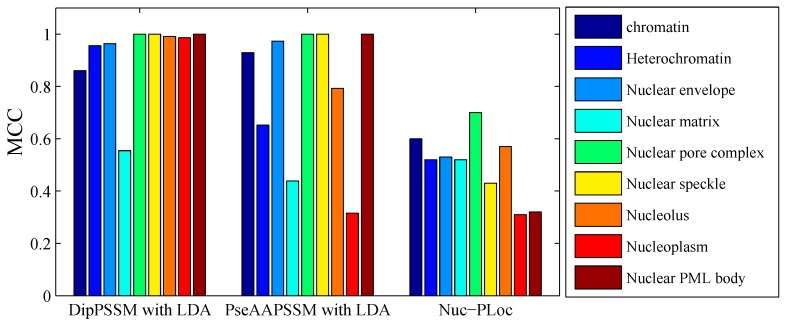
Comparison of MCC performance on Dataset 1 among our proposed methods with Nuc-PLoc.

Table 8 gives the comparison of the overall success rates on Dataset 1 among our protein sub-nuclear localization methods and the Nuc-PLoc predictor [7] with jackknife test. For each sub-nuclear location of Dataset 1, Figure 8 gives the comparison of the Matthew’s correlation coefficient (MCC) indexes [7] among our methods and Nuc-PLoc prediction. From Table 8 and Figure 8, it is clear that the success rates of our protein sub-nuclear localization predictors are much higher than that of the Nuc-PLoc.

**Table 8:** Comparison of the overall success rates by jackknife test on Dataset 1.

Algorithm	Representation	Overall Success Rate
Nuc-PLoc	Fusion of PsePSSM and PseAAC	67.4%
Our methods	DipPSSM with LDA	95.94%
	PseAAPSSM with LDA	88.1%

**Figure 9:**
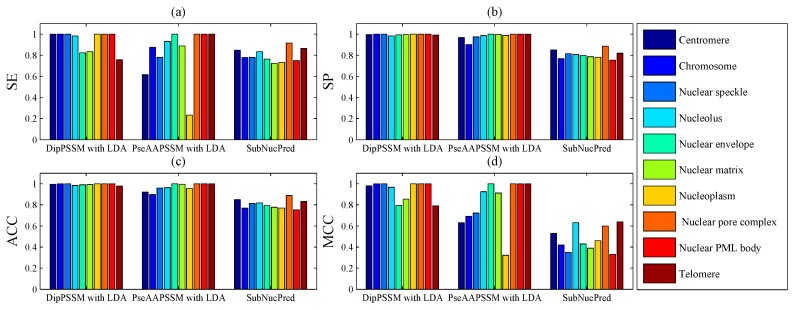
Comparison of our proposed methods with SubNucPred on Dataset 2: (**a**) Sensitivity (SE); (**b**) Specificity (SP); (**c**) Accuracy (ACC) and (**d**) Mathew’s Correlation Coeffcient (MCC).

Next, we present another comparison of our methods with SubNucPred method [17] on Dataset 2. The four indexes of sensitivity (SE), specificity (SP), accuracy (ACC) and MCC in each sub-nuclear location are calculated and shown in Figure 9, where 10-fold cross validation was used. It can be seen from Figure 9 that our methods of DipPSSM with LDA and PseAAPSSM with LDA outperform SubNucPred.

## 6. Conclusions

Following the completion of the Human Genome Project, bioscience has stepped into the era of the genome and proteome [40,41,42,43,44]. A large amount of computational methods have been presented to deal with the prediction tasks in bioscience [45,46,47,48]. The nucleus is highly organized and the largest organelle in the eukaryotic cells. Hence, managing protein sub-nuclear localization is important for mastering biological functions of the nucleus. Many current studies discuss protein sub-nuclear localization prediction [49,50]. This paper proposes a different route to identify the protein sub-nuclear localization by firstly developing two fusion representations, DipPSSM and PseAAPSSM. Then, we conduct the experiments based on the 10-fold cross validation on two datasets to certify the superiority of the proposed representations and the applicability for predicting protein sub-nuclear localization. Through the present study, we have drawn the conclusions that our fusion representations can greatly improve the success rate in predicting protein sub-nuclear localization, thereby the fusion representations can reflect more overall sequence pattern of a protein than the single one.

However, there is the difficulty of choosing proper balance factors in constructing the fusion representations. The processing method of this paper is to use genetic algorithm to produce approximate optimal values of the weight coefficients (balance factors), where we run the genetic algorithm multiple times to compute the average weight coefficient giving rise to the ideal performance. However, the time complexity of this method is high, so in the future research we will try multiple searching methods for achieving the weight coefficients.

Due to the fact that our proposed fusion representations have high dimensionality, which might result in some negative effects for KNN prediction, we employ LDA to process the representations before using KNN classifier predicts protein locations. Note that, in current pattern recognition research, many other useful data reduction methods such as kernel discriminant analysis and fuzzy LDA have emerged. How to effectively use these methods or their improved methods or other more suitable dimension reducing methods in the sub-nuclear localization field is still an open problem. In addition, it remains an interesting challenge to obtain better representations for protein sub-nuclear localization and study other machine learning classification algorithms.
